# Role of Carotid Artery Ultrasound Duplex Prior to Cardiac Surgery in Adults in Predicting Neurocognitive Complications

**DOI:** 10.7759/cureus.11211

**Published:** 2020-10-28

**Authors:** Hani N Mufti, Reham S Alshaltoni, Adel AlGahtani, Farah Jambi, Ahmed Milyani, Luis Zerpa Acosta, Saad Albugami

**Affiliations:** 1 Cardiac Surgery, King Faisal Cardiac Center, King Abdullah Medical City, Jeddah, SAU; 2 Medicine, College of Medicine, King Saud Bin Abdulaziz University for Health Sciences, Jeddah, SAU; 3 Medicine, College of Medicine, Batterjee Medical College, Jeddah, SAU; 4 Medicine, College of Medicine, Al-Maarefa University, Jeddah, SAU; 5 Medicine, College of Medicine, Ibn Sina National College, Jeddah, SAU; 6 Cardiac Surgery, King Abdulaziz Medical City, Riyadh, SAU; 7 Medicine, King Faisal Cardiac Center, King Abdullah Medical City, Jeddah, SAU; 8 Cardiology, King Faisal Cardiac Center, King Abdullah Medical City, Jeddah, SAU

**Keywords:** neurocognitive complications, carotid duplex ultrasound, cardiac surgery, outcomes

## Abstract

Background

Neurocognitive complications (NCCs) after cardiac surgery are one of the most devastating complications. Significant internal carotid artery stenosis is assumed to be a predictor of NCCs. Carotid duplex ultrasound (DUS) is a non-invasive imaging study that remains the modality of choice and is routinely used in many centers for screening adult cardiac surgery patients prior to surgery. This study aims to assess the utility of preoperative carotid DUS in the prediction of NCCs in adult patients undergoing cardiac surgery in our center.

Methods

We retrospectively reviewed the medical records of patients who underwent coronary artery bypass graft (CABG), valvular or combined surgery, at King Faisal Cardiac Center in Jeddah between January 2017 and December 2018 (n = 229). The preoperative carotid DUS findings were evaluated. Risk factors associated with NCC were analyzed.

Results

Over the study period, a total of 229 patients underwent 233 procedures. Median age was 60 years (interquartile range [IQR] = 51-67 years), of whom 71% were males. Out of the diabetic patients, 67% had an HbA1C level above 7% pre-operatively. Carotid DUS was performed on 63% of patients, but only 6.9% developed a post-operative NCC. Patients who were actively smoking were more likely to develop NCC compared to nonsmokers or ex-smokers (14.7% vs 4.6%; p = 0.02), with an odds ratio of 3.6 (95% CI = 1.2-10.5). Patients who developed NCC had a significantly higher median intensive care length of stay (7 vs. 5 days; p = 0.05).

Conclusions

Although international guidelines clearly define which patient should get preoperative carotid DUS screening, the level of evidence is low. Based on our findings, preoperative routine use of carotid DUS prior to cardiac surgery has low utility in predicting NCC. We recommend a more tailored approach based on signs, symptoms, and high-risk features to optimize the utilization of resources, avoid unwarranted delays, and personalize patient care.

## Introduction

Based on the latest World Health Organization (WHO) latest report on the top 10 causes of death, cardiovascular and cerebrovascular disease are still accounting for almost 30% of all death across the globe [1]. Although cancer is of a major concern in high-income countries, ischemic heart disease (IHD) and stroke are still the number one causes of death in middle- and low-income countries [2]. Despite increasing complexity of patients undergoing cardiac surgery, there is a decrease of associated morbidly and mortality [3-6]. Unfortunately, neurocognitive complications (NCCs) are one of the most devastating complications after any procedure and especially cardiac surgery [3-6]. A lot of patients who undergo cardiac surgery have major chronic comorbidities (diabetes mellitus [DM], hypertension [HTN], renal disease, dyslipidemia [DLP], peripheral vascular disease, etc.) that put them at higher risk of developing NCCs [4-11]. Most adult patients who undergo cardiac surgery have some element of peripheral vascular disease. One of the major concerns and risk factors for developing NCCs after cardiac surgery is the presence of significant internal carotid artery (ICA) stenosis [5,6,9,10,12,13]. The fact that the Saudi population has a higher prevalence of DM, smoking, and overweight [14] put them at higher risk of having ICA stenosis, which might increase their risk of developing NCCs after cardiac surgery. Although some patients might have significant bilateral ICA stenosis but be totally asymptomatic, others might have minimal or moderate pathology and develop symptoms [12,15,16]. Carotid artery ultrasound duplex assessment is non-invasive and easy to perform, although it requires high technical skills and is not really specific [13,15,17]. Studies regarding the routine screening of ICA stenosis and the use of carotid artery ultrasound duplex in all adult patients undergoing cardiac surgery have conflicting opinions [7,8,12,13,15-20]. A very limited number of studies are conducted in patients who undergo cardiac surgery in Saudi Arabia [13].

In this study, we intended to describe the incidence of NCCs (mainly stroke, delirium, and seizures) and judge the utility of routine use of preoperative carotid duplex ultrasound in predicting NCCs after cardiac surgery in adult patients in a single center between 2017 and 2018.

## Materials and methods

This single-center retrospective cohort chart review study included 229 adult patients who underwent cardiac surgery at King Faisal Cardiac Center (KFCC), King Abdulaziz Medical City, Ministry of National Guard Health Affairs, Jeddah, Saudi Arabia, from January 2017 to December 2018. The center was established in early 2014, and the first cardiac surgery was performed in May 2014. Around 120 adult cardiac surgeries are performed per annum. Patients were included if they were >18 years of age and underwent any of the following procedures: isolated coronary artery bypass graft (CABG) surgery, isolated valve surgery, or a combined CABG and valve procedure. Patients were identified using the KFCC cardiac surgery database.

Preoperative data collected included patient demographics such as age, gender, weight, height, and body mass index (BMI); co-morbidities such as IHD, DM, HTN, DLP, chronic kidney disease (CKD), pre-operative dialysis, smoking, chronic obstructive pulmonary disease, stroke, stroke with other co-morbidities; detailed data regarding hospital stay and invasive procedures before surgical intervention; preoperative risk factors using European System for Cardiac Operative Risk Evaluation II (EUROScore II) and the Society of Thoracic Surgeons (STS) definitions; glycated hemoglobin (HbA1C) when available; preoperative ejection fraction (EF); and pre-operative laboratory work and diagnosis. Reports of patient who underwent pre-operative carotid duplex ultrasound (DUS) were reviewed, and details were recorded.

Intra-operative data included the type of operation and procedures performed, operation duration, bypass use and duration, and administration of blood products. Postoperative data included laboratory work, blood transfusion, surgical site infection (SSI), SSI location, SSI type, mortality, and other complications. NCCs were defied as any of the following occurring after surgery: stroke, delirium, or seizures. Stroke was defined as the presence of new focal neurologic deficits after surgery that persists for more than 24 hours [3]. Delirium is defined as an acute disturbance in attention that occurs over a short period of time and is accompanied by an acute decline in cognition that cannot be accounted for by a pre-existing or evolving neurocognitive disorder such as dementia or stroke [3]. Seizures were defined as epileptic seizures associated with either generalized tonic-clonic seizures or focal abnormal repetitive movements with or without decrease of level of consciousness that was not present before surgery [21,22].

Data were obtained from patients’ hospital electronic medical record and electronic database. The electronic health record available for routine work at our hospital present data from all hospitals, primary care clinics, and laboratories belonging to the same health organization. Thus, data on background conditions, previous hospitalizations, post-discharge follow-up with hospital or clinic notes, radiological studies, surgical procedures, and readmissions were available if the case presented at our health care organization. The study was conducted in accordance with the requirements of the local ethics committee with full institutional review board approval from King Abdullah International Medical Research Center (KAIMRC).

Statistical analysis

All statistical analyses were performed using R software, version 3.6.1 (R Foundation for Statistical Computing, Vienna, Austria). Pre-, intra-, and postoperative characteristics of patients who experienced an NCC postoperatively were compared to patients who did not. The utility of DUS as a screening tool for predicting NCC was assessed.

The mean and standard deviation were used for continuous variables that had a normal distribution and were compared using the two-sample t-test or Welch two-sample t-test if the two groups had unequal variance. Continuous variable that were not normally distributed were reported using the median and interquartile range and were compared using the Wilcoxon rank sum test. Categorical variables were reported as frequencies and percentages and were analyzed using the chi-square or Fisher’s exact test as appropriate. The Kruskal-Wallis test was used for ordinal attributes. Univariate and bivariate analyses were performed to identify risk factors for the development of NCCs after cardiac surgery, especially pre-operative DUS screening, the severity of carotid artery stenosis, and the influence of pre-operative DUS on NCCs. All statistical tests were two-tailed, and p-values < 0.05 were considered significant.

## Results

Demographics and clinical characteristics

Between January 2017 and December 2018, 229 patients underwent cardiac surgical procedures at KFCC. Of these, 71% (n = 162) were males. Median age was 61 years (interquartile range [IQR] = 51-67 years) and BMI was 29 kg/m^2^ (IQR = 25.8-32.5). Almost 66% (n = 150) had DM and 35% (n = 81) were on insulin. Most patients were hypertensive (~76%) and had a history of IHD (~75%). Only 12.7% of patients had a history of CKD, and only 4.4% of patients were on hemodialysis prior to surgery. 

More than two-thirds of patients had an EF above 40% (~78%) prior to surgery, and almost half of patients had a pre-operative HbA1c > 7% (47%). Three-quarters of patients had elective surgery (75%), and 60% of them underwent CABG. Median EUROScore II was 1.8 (IQR = 1.1-2.7), and the STS adult cardiac surgery risk score was 1 (IQR = 0.5-2) (Table 1).

**Table 1:** Pre-Operative Characteristics BMI, body mass index; CKD, chronic kidney disease; EUROScore II, European System for Cardiac Operative Risk Evaluation II; HbA1C, hemoglobin A1C, which is glycated hemoglobin; IHD, ischemic heart disease; IQR, interquartile range; STS, Society of Thoracic Surgeons

Patient characteristics	Patients, n = 229
Age in years, median (IQR)	61 (51-67)
Male gender, n (%)	162 (70.7)
BMI (kg/m^2^), median (IQR)	29 (25.8-32.5)
Hypertension, n (%)	175 (76.4)
Diabetes mellitus, n (%)	150 (65.5)
IHD, n (%)	172 (75.1)
Dyslipidemia, n (%)	185 (80.8)
CKD, n (%)	29 (12.7)
Pre-operative dialysis, n (%)	10 (4.4)
Smoking, n (%)	
Active	49 (21.4)
Ex-smoker	437 (16.2)
Non-smoker	143 (62.5)
Chronic obstructive pulmonary disease, n (%)	9 (3.9)
Pre-operative stroke, n (%)	17 (7.4)
Pre-operative ejection fraction, n (%)	
<25 %	4 (1.75)
25-40 %	34 (14.85)
41-55 %	108 (47.15)
>55%	71 (31)
Unknown	12 (5.2)
Pre-operative HbA1C, n (%)	
≤7 %	90 (39.3)
>7 %	108 (47.2)
Unknown	9 (3.9)
Pre-operative EUROScore II category, n (%)	
<5 %	203 (88.7)
≥5 %	14 (6.1)
Not applicable	12 (5.2)
Pre-operative STS mortality score category, n (%)	
<5 %	207 (90.4)
≥5 %	10 (4.4)
Not applicable	12 (5.2)

Almost 64% of patients in our cohort underwent pre-operative assessment of their carotid arteries using DUS. Only one patient had a right ICA stenosis more than 75%, and two patients had right ICA stenosis more than 75%. None of the patients had bilateral ICA stenosis more than 75%. Only 21 patients had a right ICA stenosis more than 50%, and 18 patients had right ICA stenosis more than 50%. Five patients had bilateral ICA stenosis more than 50%, and no patient had more than 75% bilateral ICA stenosis (Table 2).

**Table 2:** Carotid DUS Findings DUS, duplex ultrasound; ICA, internal carotid artery

Patient characteristics	Patients, n = 229
Pre-operative carotid DUS, yes, n (%)	146 (63.8)
Pre-operative carotid DUS - right ICA stenosis, n (%)	
<25%	13 (5.7)
25-50%	48 (20.9)
50-75%	20 (8.7)
>75%	1 (0.4)
Unknown (not mentioned)	64 (27.9)
Not done	83 (36.2)
Pre-operative carotid DUS - right ICA stenosis more than 50%, n (%)	21 (9.2)
Pre-operative carotid DUS - left ICA stenosis, n (%)	
<25 %	11 (4.8)
25-50%	48 (20.9)
50-75%	16 (7)
>75%	2 (0.9)
Unknown (not mentioned)	69 (30.1)
Not done	83 (36.2)
Pre-operative carotid DUS - left ICA stenosis more than 50%, n (%)	18 (7.9)
Pre-operative carotid DUS - bilateral ICA stenosis more than 50%, n (%)	5 (2.2)
Pre-operative carotid DUS - bilateral ICA stenosis more than 75%, n (%)	0

The median length of stay in the intensive care unit was 5 days (range: 4-7 days), and hospital length of say was 14 days (IQR = 10-20 days). Over half of the patients received a post-operative blood transfusion, 5.7% required dialysis after surgery, and only 3.5% of patients suffered a post-operative stroke. Post-operative mortality was 6.6% (Table 3).

**Table 3:** Intra- and Post-Operative Characteristics AVR, aortic valve replacement; CABG, coronary artery bypass grafting; CT, computed tomography; ICU, intensive care unit; IQR, interquartile range; MVR, mitral valve replacement

Patient characteristics	Patients, n = 229
Procedure status, n (%)	
Elective	172 (75)
In-house urgent	39 (17)
Emergency	13 (5.6)
Unknown	5 (2.2)
Procedure, n (%)	
CABG	138 (60.3)
AVR	14 (6.1)
MVR	16 (7)
Combined	33 (14.4)
Other	28 (12.2)
Cardio-pulmonary bypass time (minutes)	
Median (IQR)	120.5 (95-165.8)
Aortic cross-clamp time (minutes)	
Median (IQR)	79 (59.3-128.3)
Length of stay in the ICU (days)	
Median (IQR)	5 (4-7)
Length of stay in the hospital (days)	
Median (IQR)	14 (10-20)
Post-operative blood transfusion, n (%)	122 (53.3)
Post-operative dialysis, n (%)	13 (5.7)
Post-operative stroke, n (%)	8 (3.5)
Post-operative delirium, n (%)	9 (3.9)
Post-operative seizures, n (%)	3 (1.3)
Post-operative CT of the brain, n (%)	15 (6.6)
Post-operative neurocognitive complication, n (%)	16 (7)
Post-operative surgical site infection, n (%)	67 (29.3)
Post-operative mortality, n (%)	15 (6.6)

NCC after cardiac surgery

**Figure 1:**
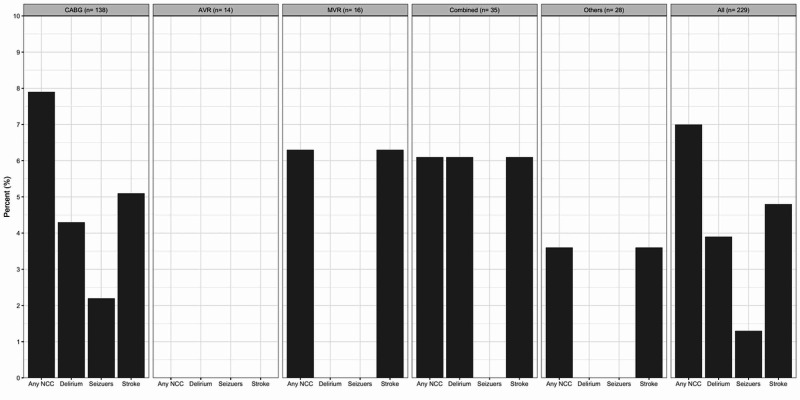
NCC by Procedure AVR, aortic valve replacement; CABG, coronary artery bypass grafting; MVR, mitral valve replacement; NCC, neurocognitive complication

Out of 229 adult patients, 16 (7%) patients developed a suffered from a neurocognitive complication after cardiac surgery. Out of the 16 patients, 9 developed delirium, 8 suffered a stroke, 3 had seizures, 3 experienced stroke and delirium together, and only 1 developed seizure and was diagnosed with a stroke. Stroke was the most common NCC across all procedures (Figure 1). Brain computed tomography (CT) scan was performed for 50% of these patients, and 75% of these patients required a post-operative blood transfusion. Most of the patients who suffered from a neurocognitive complication were males (94%), and 56% of them were over the age of 60 years. Median length of stay in the ICU was significantly longer in patients who suffered an NCC compared to the ones who did not (7 vs 5 days; p = 0.05). Blood transfusion after surgery was higher in the NCC group (81% vs 51%, p = 0.02). Post-operative mortality was significantly higher in patients with NCC (27% vs 5.2%; p = 0.014).

Risk factors for NCC after cardiac surgery

Bivariate analysis was used to assess the relationship between several variables and patients who suffered an NCC compared to the ones who did not. This identified three possible predictors of NCC after cardiac surgery in adult patients with p < 0.05 (male gender, pre-operative history of CKD, pre-operative EF < 40%, and active smoking) (Table 4). Interestingly, age above 60 years, history of stroke before surgery, the use of DUS before surgery, even the percentage of stenosis in the ICA, procedure being other than elective, and procedure other than CABG were not found to be associated with post-operative NCC.

**Table 4:** Bivariate Analysis Showing Significant Predictors of NCC CABG, coronary artery bypass grafting; CKD, chronic kidney disease; DUS, duplex ultrasound; ICA, internal carotid artery; IQR, interquartile range; NCC, neurocognitive complication; STS, Society of Thoracic Surgeons *Statistically significant with p < 0.05.

Patient characteristics	Total patients (n = 229)	NCC, yes (n = 16 [7%])	NCC, no (n = 213 [93%])	p-Value
Age ≥ 60 years, n (%)	124 (54.2)	9 (56.2)	115 (54)	0.9
Male gender, n (%)	162 (70.7)	15 (93.8)	147 (69)	0.036*
History of CKD, n (%)	29 (12.7)	5 (31.2)	24 (11.3)	0.021*
Pre-operative stroke, n (%)	17 (7.4)	3 (18.8)	14 (6.6)	0.07
Smoking active, n (%)	49 (21.4)	8 (50)	41 (19.2)	0.004*
Pre-operative ejection fraction < 40%, n (%)	38 (16.6)	6 (37.5)	32 (15)	0.02*
Pre-operative carotid DUS - yes, n (%)	146 (63.8)	9 (56.2)	137 (64.3)	0.52
Pre-operative carotid DUS - right ICA stenosis more than 50%, n (%)	21 (9.2)	0	21 (15.3)	0.36
Pre-operative carotid DUS - left ICA stenosis more than 50%, n (%)	18 (7.9)	2 (22.2)	16 (11.7)	0.31
Pre-operative carotid DUS - bilateral ICA stenosis more than 50%, n (%)	5 (3.4)	0	5 (2.4)	0.99
Pre-operative STS stroke score, median (IQR)	1.2 (0.8-1.8)	1.3 (1-2.1)	1.2 (0.8-1.8)	0.21
Procedure status elective, n (%)	172 (75.1)	14 (87.5)	158 (74.2)	0.24
Procedure only CABG, n (%)	138 (60.3)	11 (68.8)	127 (59.6)	0.47

Outcomes of patients who develop NCC after cardiac surgery

Out of the 16 patients who developed NCC, 8 suffered a stroke, 9 experienced delirium, and 3 had seizures, with almost 69% (11 patients) of events occurring in patients who underwent CABG (Table 4). Median length of stay in the ICU was marginally longer in patients who developed an NCC (7 vs 5 days; p = 0.05). Blood transfusion was significantly higher in the NCC group (81 vs 51%; p = 0.02), and post-operative mortality was high (27 vs 5%; p = 0.014). Patients who suffer an NCC had seven times the odds of an in-hospital mortality (95% CI = 1.7-25.1; p = 0.004).

Out of the eight patients who developed post-operative stroke, five had pre-operative carotid DUS, with only one having a left ICA stenosis of 50-75%. Seven out of the eight patients who developed post-operative stroke underwent a post-operative CT of the brain, which showed small bilateral strokes that are consistent with cardio-embolic source rather than right-sided ischemia, even in the patient who had left ICA stenosis of 50-75%. Two patients had left posterior inferior cerebellar artery (PICA) ischemic finding with normal carotid DUS. One patient had right middle cerebral artery ischemia who did not have a carotid DUS before or after his stroke. Two of the stroke patients developed pneumonia due to pseudomonas and one due to *Escherichia coli*. One patient developed urinary tract infection (UTI) due to *E. coli*. Two patients with stroke had superficial sternal wound infection with gram-negative organisms (*Klebsiella pneumoniae *and *Pseudomonas aeruginosa*) that required wound care and antibiotics.

Of the nine patients who developed delirium, only one had a left ICA stenosis > 75% but had no stroke and did not undergo a CT of the brain because he improved within 48 hours. Three out of the nine patients had delirium and stroke, of which two had left PICA and one had bilateral ischemic findings on CT of the brain. Two of the patients with delirium had pneumonia (one with *E. coli *and one with *Citrobacter*). None of the patients who developed delirium had UTI or sepsis. One patient with delirium had deep sternal wound infection with *Staphylococcus aureus* that required debridement.

Three patients developed seizures and underwent a post-operative CT of the brain. The brain CTs of two out of the three patients were normal, whereas one patient had a left posterior inter-cerebral artery (PICA) related ischemia. The three patients who developed seizures underwent CT of the brain, out of which only one had left PICA ischemia and suffered from stroke and delirium. This patient had pneumonia due to *E. coli*. None of the seizure patients had post-operative UTI or wound infection.

## Discussion

Compared to other post-operative complications, NCCs remain the most devastating complications after cardiac surgery. In our study, the incidence of NCCs after cardiac surgery was quite low (7%), with stroke being the most common one (3.5% of the whole population). This is consistent with the published literature, with stroke ranging between 2% and 5% [3,4,6,9,12,19]. Although the incidence of delirium was very low in our cohort (3.8%), most of these patients would have been from the agitated delirium group [3]. With hypoactive delirium being the most common form of delirium after cardiac surgery, we could have missed a large proportion of those patients who have higher incidence of cognitive decline and worse quality of life after discharge [23-29].

Several studies have identified factors that are associated stroke after cardiac surgery (e.g., old age, complexity of the procedure, history of pre-operative stroke, peripheral vascular disease) [3-5,7-9]. In our study, male gender, pre-operative history of CKD, EF < 40% before surgery, and active smoking were identified as strong predictors for NCCs after cardiac surgery. In contrast to other studies, history of stroke was not found to be a predictor for NCCs after cardiac surgery in our cohort [4,6-8].

As coronary artery disease is a systematic disease, it is assumed that it is associated with extra-cranial carotid artery disease [5,9,12,15,16,18,20]. Carotid duplex ultrasound is an easy non-invasive screening test that can be used to identify patients with significant ICA stenosis; it has a moderate sensitivity and low specificity in identifying patients who are at risk of developing stroke after cardiac surgery [5,9,12,15,16,18,20]. In a statement by the American Heart Association (AHA) 2011 CABG guidelines, it was stated that the use of DUS was reasonable in only high-risk patients (level of evidence = C; class IIa) [7]. Stroke after cardiac surgery is multifactorial and can be attributed to several causes (e.g., thrombus in the heart or aorta, manipulation of the aorta, arrhythmia, inflammation, the cardiopulmonary bypass machine) [4-6,8]. According to the European Society of Cardiology (ESC) and the European Association for Cardio-Thoracic Surgery (EACTS) 2018 Guidelines on myocardial revascularization, there is insufficient evidence to support the association between ICA stenosis and stroke after cardiac surgery [8], and prophylactic carotid endarterectomy showed no short-term benefit in preventing post-operative stroke [8,17]. The 2018 ESC/EACTS guidelines on myocardial revascularization recommends DUS based on the pre-operative history of recent stroke (less than six months) (level B, class I). For patients with a history of remote stroke (more than six months), carotid DUS may be considered before CABG in the following cases: age >70 years, multivessel coronary artery disease, peripheral vascular disease (PVD), or carotid bruit on physical examination [8]. Several authors have investigated the utility of routine use of DUS in patients, and most of them found that there is a low yield of routine screening and that a selective screening approach in high-risk patients was more clinically relevant and cost-effective [12,15,16,18,20].

In a local study by Arifi et al., they retrospectively reviewed 3,150 adult patients who underwent cardiac surgery in a single institution over a period of 10 years and had a pre-operative DUS [9]. They reported significant ICA stenosis of >75% in 6.6% of patients. Patients with significant ICA stenosis of >75% were more likely to be diabetic and hypertensive and have a history of stroke and PVD. It was also was associated with major morbidity, with 4.3% of these patients sustaining a post-operative stroke, and a higher incidence of all-cause 30-day mortality. Another study by Waheed et al. [13] investigated 178 patients who underwent CABG in a single center over a three-year period. In this study, the incidence of ICA stenosis > 70% was 6.2% and stroke occurred in 4.5% of patients. They determined that advanced age, ICA stenosis, and multi-vessel coronary arteries disease were linked to post-CABG adverse events.

Interestingly, in our study, neither advanced age nor ICA stenosis was associated with NCCs after cardiac surgery in adults. However, patients who sustained an NCC after cardiac surgery were more likely to have a length of stay in the ICU of more than 8 days, required more blood transfusion, and had higher all-cause in-hospital mortality. The incidence of post-operative stroke in our cohort and especially in the CABG subgroup was consistent with other published literature [3,4,6,9,12,13,16,18,20].

Limitations

Some of the limitations of this work include the latent bias of retrospective studies that is based on observational data that is based on chart abstraction. The quality of the acquired data might profoundly impact the interpretation. Another limitation is the small sample size and the study being a single-center study, which might limit the generalization of the results. Patients demographics might differ based on the geographical location and catchment area of the treating center. Patient who did not follow-up in our center were also not included in our cohort as we do not know if they developed a sequela of their NCC. Fortunately, we do have a small number of events. Finally, there might be a good proportion of patients who developed hypoactive delirium and were missed because a standard delirium assessment tool such as the Confusion Assessment Method (CAM) is not being used routinely in our ICU.

## Conclusions

NCCs after cardiac surgery is not that common, yet they are devastating. Patients undergo cardiac surgery with the main goal to improve their quality of life. With increasing complexity of patients undergoing cardiac surgery, the number of NCC is expected to increase. Although international guidelines clearly define which patient should undergo preoperative carotid DUS screening, the level of evidence is low. Based on our findings, preoperative routine use of carotid DUS prior to cardiac surgery has low utility in predicting NCC. We recommend a more tailored approach based on signs, symptoms, and high-risk features to optimize the utilization of resources, avoid unwarranted delays, and personalize patient care.
